# A Novel Biomarker Driving Poor-Prognosis Liver Cancer: Overexpression of the Mitochondrial Calcium Gatekeepers

**DOI:** 10.3390/biomedicines8110451

**Published:** 2020-10-24

**Authors:** Chia-Jung Li, Hung-Yu Lin, Chih-Jan Ko, Ji-Ching Lai, Pei-Yi Chu

**Affiliations:** 1 Department of Obstetrics and Gynecology, Kaohsiung Veterans General Hospital, Kaohsiung 813, Taiwan; nigel6761@gmail.com; 2 Institute of BioPharmaceutical Sciences, National Sun Yat-sen University, Kaohsiung 804, Taiwan; 3 Research Assistant Center, Show Chwan Memorial Hospital, Changhua 500, Taiwan; linhungyu700218@gmail.com; 4 Department of General Surgery, Changhua Christian Hospital, Changhua 500, Taiwan; 91681@cch.org.tw; 5 School of Medicine, Kaohsiung Medical University, Kaohsiung 807, Taiwan; 6 Department of Medical Research and Development, Chang Bing Show Chwan Memorial Hospital, Changhua 505, Taiwan; jichinglai@gmail.com; 7 School of Medicine, College of Medicine, Fu Jen Catholic University, New Taipei 242, Taiwan; 8 Department of Pathology, Show Chwan Memorial Hospital, Changhua 500, Taiwan; 9 Department of Health Food, Chung Chou University of Science and Technology, Changhua 510, Taiwan; 10 National Institute of Cancer Research, National Health Research Institutes, Tainan 704, Taiwan

**Keywords:** hepatocellular carcinoma, mitochondrial Ca^2+^ uptake, CREB, MCU, MICU1, MICU2, prognosis, prediction

## Abstract

Several studies have indicated the biological role of mitochondrial Ca^2+^ uptake in cancer pathophysiology; however, its implications in predicting the prognosis of hepatocellular carcinoma (HCC) are not yet fully understood. Here, we collected tumor specimens and adjacent normal liver tissues from 354 confirmed HCC patients and analyzed the levels of cyclic adenosine monophosphate (cAMP) responsive element binding protein 1 (CREB), mitochondrial calcium uniporter (MCU), mitochondrial calcium uptake 1 and 2 (MICU1, MICU2) using bioinformatics, qRT-PCR, and immunohistochemistry (IHC), and their relationship with clinicopathological characteristics and prognosis. HCC patients with low CREB/MICU1 and high MCU/MICU2 expression exhibited poor survival rate and prognosis in overall survival (OS) and disease-free survival (DFS) analyses. Low CREB/MICU1 and low MICU1 alone indicated poor prognosis in stage I/II and III/IV patients, respectively. In the poor differentiation/undifferentiation group, low expression of MICU1 indicated poor clinical outcomes. Low CREB/MICU1 expression suggested poor outcomes in patients with or without hepatitis B virus (HBV) infection and poor prognosis in the HCV infection group. In the non- hepatitis C virus (HCV) infection group, low MCU1 indicated a poor prognosis. Multivariate analysis demonstrated that CREB and MICU1 expression showed prognostic significance. This study demonstrates the prognostic significance of CREB, MCU, MICU1, and MICU2, in predicting HCC outcomes. Low CREB/MICU1 and high MCU/MICU2 in HCC tissues are associated with poor prognosis, thus offering a novel perspective in the clinical management for HCC patients.

## 1. Introduction

Hepatocellular carcinoma (HCC) is the sixth most common cancer and the fourth leading cause of cancer-related deaths worldwide, accounting for approximately 841,000 new cases and 782,000 deaths [1]. In 2018, HCC became the fifth most common cancer with the second-highest mortality rate worldwide [2]. Although several risk factors have been linked to HCC [3], the accurate prognostic signatures are yet to be fully elucidated.

Mitochondria are vital cell organelles serving as the intracellular power plant regulating cellular life and death, that are actively involved in cellular Ca^2+^ signaling [4]. Mitochondria accumulate Ca^2+^ and have a ubiquitous physiological and pathophysiological role in Ca^2+^ handling [5]. Ca^2+^ accumulation within the mitochondria regulates the intrinsic functions of the organelle. One of the most characteristic roles of mitochondrial Ca^2+^ uptake is in the control of metabolic activity. Three dehydrogenases of the tricarboxylic acid (TCA) cycle, namely pyruvate dehydrogenase (PDH), α-ketoglutarate, and isocitrate dehydrogenases (IDH), are activated by mitochondrial matrix Ca^2+^ [6,7]. Mitochondrial Ca^2+^ also modulates the production of reactive oxygen species (ROS) critical for carcinogenesis and drug resistance in HCC [8].

The mitochondrial Ca^2+^ uniporter (MCU) complex is the key regulator of the accumulation of mitochondrial Ca^2+^ and its homeostasis. The MCU complex is composed of the pore-forming subunit of the mitochondrial Ca^2+^ uptake channel (MCU), regulatory subunits of mitochondrial calcium uptake 1 and 2 (MICU1 and MICU2), MCUb, and essential MCU regulator (EMRE) [4]. Various studies have shown that a disturbance in mitochondrial Ca^2+^ homeostasis caused by the MCU complex has a severe impact on tumor progression [9,10]. The expression of MCU is controlled by the Ca^2+^-dependent transcription factor, cyclic adenosine monophosphate response element-binding protein (CREB), which directly interacts with the promoter of MCU to stimulate gene expression [11]. The function of CREB to regulate the proliferation of normal and cancerous liver cells has been shown previously. CREB modulates the signal coupling of cyclic adenosine monophosphate (cAMP), which constitutes an activator or repressor family, and binds to the cAMP response promoter element (CRE) in the cAMP-regulatory region. The role of the CREB family in controlling the progression of hepatocellular carcinoma has been advocated. In addition, CREB may be activated by the X protein of the hepatitis B virus, which binds to the unphosphorylated form of CREB and activates transcription [12,13,14]. Previous studies have demonstrated that mitochondrial Ca^2+^ balance plays a role in tumor pathophysiology. For instance, the upregulation of MCU has been identified in endoplasmic reticulum-negative and basal-like breast cancer [15]. Previous studies have confirmed that, in HCC, MCU gene inactivation can inhibit the metabolism of HCC cells, which in turn leads to decreased HCC cell proliferation and reduced cancer metastasis. This indicates that the regulation of mitochondrial calcium plays an important role in HCC [16]. However, the comprehensive profile of the genes involved in mitochondrial Ca^2+^ homeostasis in HCC and their prognostic relevance in patients is unknown. In this study, we used the the Cancer Genome Atlas (TCGA) database to analyze the expression, survival rate, and correlation of these genes in liver cancer. We further cross-validated the genetic differences through different liver cancer and normal liver cell lines. Finally, we recruited 354 Asian liver cancer patients (including hepatitis B virus HBV and hepatitis C virus HCV) for large-scale analysis of the mitochondrial genes CREB, MCU, MICU1, and MICU2, and used immunohistochemistry (IHC) and bioinformatics techniques to analyze their clinical characteristics to predict the prognosis of HCC.

We further studied the role of MCU and the assembly proteins MICU1 and MICU2 in the growth of HCC. CREB affects the expression of the MCU protein in HCC patients thus leading to the observed changes in mitochondrial Ca^2+^ homeostasis. Interestingly, even in the presence of activated CREB, there is an increase in the levels of MCU, MICU1, and MICU2 that significantly enhances tumor growth. These data indicate that the mitochondrial CREB–MCU axis plays a key role in the progression of liver cancer.

## 2. Patients and Methods

### 2.1. Patient Characteristics

This study included 354 patients diagnosed with HCC, in the Division of General Surgery, Department of Surgery, Changhua Christian Hospital, Taiwan, between November 2013 and September 2017. The inclusion criteria included pathologically confirmed cases of HCC with patients aged >18 years. Pregnant and/or patients who had previously undergone radiotherapy, transcatheter arterial embolism, transarterial chemoembolization chemotherapy, targeted therapy, or surgical intervention were excluded from this study. The included HCC patients underwent curative surgical resection. The detailed clinical data, pathological findings, and surgical outcomes were recorded. Following the surgical intervention, the tumor specimens were investigated and adjacent normal liver tissues were collected for IHC staining. Moreover, the duration of follow-ups, defined from the date of surgical intervention to the patient’s last visit or death, and the status of death, censorship, or lack of follow-up were recorded. All the protocols in this study, including IHC staining, were approved by the Institutional Review Board of Changhua Christian Hospital (CCH IRB number: 170909, 2017/09/09). Informed consent was obtained from all the HCC patients. This study included 354 patients (80.5% males) with a mean age of 63.2 ± 11.5 years. Analysis of severity revealed that a majority of the patients had Child-Pugh A score (Child-Pugh points) of 5.2 ± 0.7, a mean Ishak score of 3.8 ± 1.6, a mean Metavir score of 2.7 ± 1.2, and 84.2% had clinical stage I and II HCC. The mean tumor size was 33.7 ± 20.0 mm. The prevalence rates of hepatitis B and C infections were 54.0% and 31.3%, respectively. Pathological studies revealed poor differentiation/undifferentiation in 54.5% (*n* = 193) and well/moderate differentiation in 45.2% (*n* = 160) of the patients. The overall survival rate was 82.2%, and the recurrence rate was 18.4%. 36.7% (*n* = 130) of the patients received two segmental resections. The mean survival duration was 796.8 ± 422.6 days. The characteristics of all the patients included in this study are listed in Table 1.

### 2.2. Immunohistochemistry Staining and Scoring

Four antibodies used to target CREB, MCU, MICU1, and MICU2 were purchased from Biorbyt (Cambridge, UK). After tumor resection, the tissues were embedded in paraffin and cut into 4-μm thick sections. The slides were coated with poly-L-lysine and deparaffinized by rinsing with 10 mM Tris-HCl (pH 7.4) and 150 mM sodium chloride. Next, all the slides were treated with methanol and 3% hydrogen peroxide and placed in a heating chamber with a temperature of less than 100 °C in 10 mM citrate buffer for 30 min. The slides were then divided into four groups, and each group was treated with one of the four antibody solutions, CREB (1:100), MCU (1:100), MICU1 (1:150), and MICU2 (1:400), for 1 h. The control samples were processed without any primary antibody. After incubation, the slides were washed with phosphate-buffered saline and analyzed using EnVision Detection Systems, Peroxidase/ 3, 3 -diaminobenzidine (DAB), Rabbit/Mouse kit (Dako, Glostrup, Denmark). All the slides were investigated under a microscope (BX50, OLYMPUS, Tokyo, Japan) and evaluated by two physicians and the digital pathological biopsy scanning services from Biotechnology Corporation. IHC analyses included a scoring system involving two aspects, namely, staining intensity and percentage of positive cells. The staining intensity was divided into four grades, including 0 (no expression), 1 (weak expression), 2 (moderate expression), and 3 (strong expression). The total score ranged from 0 to 300, calculated as staining intensity × percentage of positively labeled cells.

### 2.3. Multi-Omics Analysis

Gene Expression Profile Analysis Interactive Analysis (GEPIA) is a user-friendly interactive network database that can be linked and analyzed with other databases (TCGA and GTEx). Using GEPIA, we analyzed in 9736 tumors and 8587 normal tissues [17]. Further, using UALCAN, a database that can efficiently find RNAseq data in TCGA and perform gene expression and survival analyses, we analyzed the expression of CREB1 in different clinical stages of ovarian cancer [18]. GeneMANIA is an open website for building protein–protein interaction (PPI) networks and predicting gene function. This website analyzes gene(s) lists using bioinformatics techniques, including gene co-expression, physical interaction, gene co-location, gene enrichment analysis, and website prediction.

### 2.4. RNA Extraction and Real-Time PCR

The total RNA was extracted with the EasyPrep Total RNA Kit (Biotools, Taipei, Taiwan). A total of 1 μg of RNA was reverse-transcribed with a ToolScript MMLV RT kit (Biotools, Taipei, Taiwan) for cDNA synthesis. Real-time polymerase chain reaction (PCR) was carried out using a StepOnePlusTM system (Applied Biosystems, Foster City, CA, USA) with TOOLS 2X SYBR qPCR Mix (Biotools, Taipei, Taiwan). The expression levels of all the genes in cells were normalized to the internal control GAPDH gene. All the samples with a coefficient of variation for Ct value >1% were retested.

### 2.5. Statistical Analysis

All data were presented as mean ± standard deviation (SD) or case number (%). The correlation between the clinicopathological parameters and the expressions of the four genes was analyzed using the chi-square or Fisher exact tests for categorical variables and paired-sample *t*-test for continuous variables, using the SPSS software (Version 13.0; SPSS Inc., Chicago, IL, USA). The Spearman rank correlation test was used to analyze the correlation results of the expression of the four biomolecules. In this study, the endpoints were overall survival (OS) and disease-free survival (DFS). The results of the univariable analysis of the variables and survival data were obtained using the Kaplan–Meier method with the log-rank test. The relationship between the variables and survival data was analyzed via Cox’s proportional hazards regression model. Statistical significance was defined as a *p*-Value <0.05.

## 3. Results

### 3.1. CREB, MCU, MICU1, and MICU2 Levels Were Greatly Upregulated in HCC Patients

**Figure 1:**
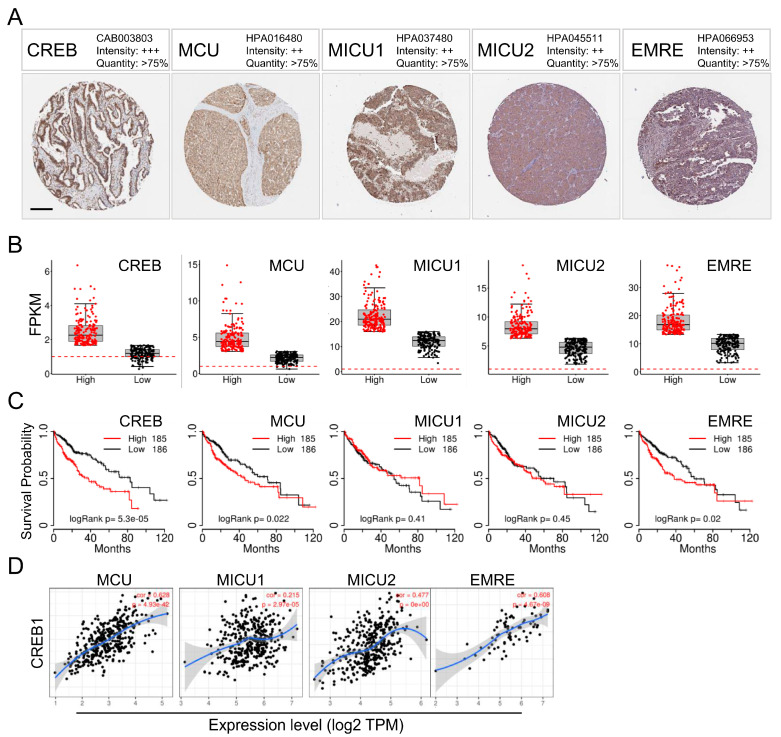
CREB, MCU, MICU1, and MICU2 expression in HCC and its effect on prognosis. (**A**) Representative images of CREB, MCU, MICU1, and MICU2 immunohistochemistry (IHC) staining in HCC from the Human Protein Atlas (HPA) (**B**) Plots chart showing higher CREB, MCU, MICU1, and MICU2 expression in HCC patients. Data were obtained from TCGA. (**C**) Kaplan–Meier curves of overall survival in HCC patients. Survival data were obtained from TCGA. CREB expression was positively correlated with the expression of MCU, MICU1, and MICU2 in TCGA dataset (**D**). Scale bars 200 μm.

First, we used a tissue microarray (TMA) from the Human Protein Atlas (HPA) to analyze the levels of the CREB, MCU, MICU1, and MICU2 proteins. In HCC patients, we found high-grade nuclear staining for CREB in HCC patients and moderate MCU staining; MICU1 and MICU2 individually showed moderate staining (Figure 1A). Next, we determined the transcriptional expression of the target genes differentially expressed between HCC and normal tissues in TCGA. The mRNA levels of CREB, MCU, MICU1, and MICU2 were found to be significantly increased in liver cancer, indicating that these proteins may have potential carcinogenic effects (Figure 1B). In addition, HCC patients with high levels of CREB and MCU mRNA expression had low overall survival (Figure 1C). Furthermore, CREB expression was found to be correlated with MCU (*p* = 4.93 × 10^−42^, R = 0.628) and MICU1 (*p* = 2.97 × 10^−5^, R = 0.215) expression, but not with MICU2 expression (*p* = 0 × 10^0^, R = 0.477) in the TCGA-COAD dataset (Figure 1D). Taken together, these data highlight the association between MCU/MICU1 expression and CREB activation in HCC.

### 3.2. CREB Transcription Factor Is an Upstream Regulatory in Liver Cancer Cells

**Figure 2:**
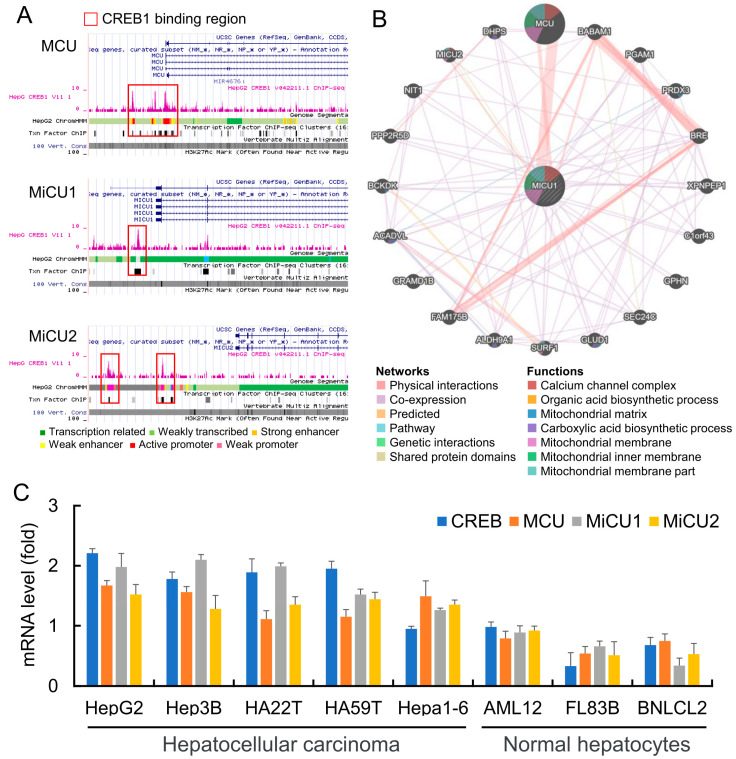
CREB was highly expressed and closely correlated with MCU, MICU1, and MICU2 in HCC. (**A**) Scheme of the human genomic region encompassing the MCU, MICU1, and MICU2 promoter. The pink peaks represent the CREB-binding regions, according to ENCODE. The red square shows the stronger binding region of each promoter. (**B**) Functional enrichment and signal pathway analysis of differentially expressed genes in immunotype B vs. immunotype A subgroup. (**B**) Genes associated with MICU1 are represented by circles. Networks were divided into 6 parts. Physical interaction among them was 67.6%, co-expression was 13.5%, predicted was 6.4%, co-localization was 6.2%, pathway was 4.4%, gene interaction was 1.4%. (**C**) RT-PCR was used to detect the expression levels of different liver cancer cells and normal liver cells, and glyceraldehyde-3-phosphate dehydrogenase (GAPDH) was used as an internal control.

Next, we explored the upstream regulators that may potentially mediate the increased mitochondrial relative protein expression. Analysis of the array expression database revealed that CREB1 has a strong binding site in the upstream promoter region of MCU and MICU2 in liver cancer (HepG2) cells. Previously published sequencing data showed that CREB1 can directly bind to the MCU and MICU2 promoters (Figure 2A). Furthermore, the protein interaction network revealed the correlation between the MICU1 genes. The gene set rich in MICU1 was found to be responsible for organic acid and carboxylic acid biosynthesis and protein assembly of the Ca^2+^ channel complex (Figure 2B). Next, we analyzed the endogenous levels of CREB, MCU, MICU1, and MICU2 in liver cancer cells and normal liver cell lines, and the results showed that the mRNA expression levels of CREB, MCU, MICU1, and MICU2 in liver cancer cell lines were higher than that in normal liver cells. (Figure 2C).

### 3.3. Association of the CREB, MCU, MICU1 and MICU2 Protein Levels with Hepatocellular Tumorigenesis and Clinicopathological Outcomes

**Figure 3:**
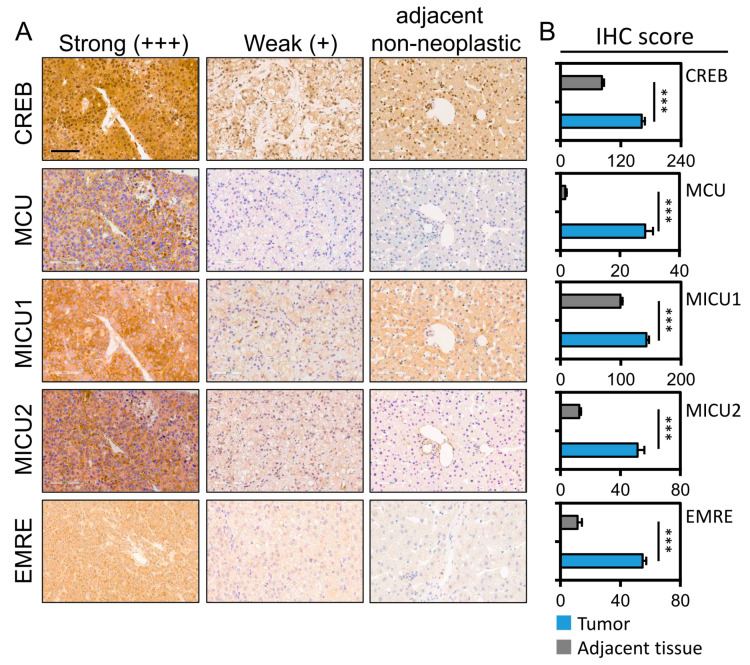
Immunoreactivity of CREB, MCU, MICU1, and MICU2 in hepatocellular carcinoma (HCC). (**A**) The representative photomicrographs of CREB, MCU, MICU1, and MICU2 expression for adjacent non-neoplastic, weak (+), and strong (+++) staining in HCC tissues. (**B**) The IHC scores of CREB, MCU, MICU1 and MICU2 expression in hepatocellular carcinoma tissue and matched adjacent normal liver tissue. *** *p* < 0.001. Scale bars 200 μm.

The IHC data of four mitochondrial gene expressions in HCC and adjacent normal tissues are shown in Figure 3A. The CREB, MCU, MICU1, and MICU2 expressions were significantly high in normal tissues. The expression of all the four mitochondrial proteins was significantly higher with higher IHC scores in the tumor tissues than in the normal liver tissues (median IHC score of CREB: 158.3 vs. 90.0, *p* < 0.001; MCU: 27.1 vs. 0.2, *p* < 0.001; MICU1: 139.4 vs. 97.8, *p* < 0.001; MICU2: 49.0 vs. 9.2, *p* < 0.001; Figure 3B). Based on the IHC scores in the tumor group, all the patients were further categorized into low and high expression groups using the median IHC score of the four gene expressions as the cutoff value (Figure 3B). The statistically high expression of MCU, MICU1, and MICU2 in HCC was associated with a higher clinical stage and poor-differentiation histologic grade. High expression of MCU and MICU1 were also statistically associated with a poor survival rate. The analysis of the cytoplasmic form of CREB revealed that high expression was associated with a good prognosis. In addition, high expression of MCU was associated with a high level of hepatitis C infection. The correlation between the expression of the four genes was significant, as shown in Table 2.

### 3.4. Mitochondrial Gene Expression and Survival Analysis

**Figure 4:**
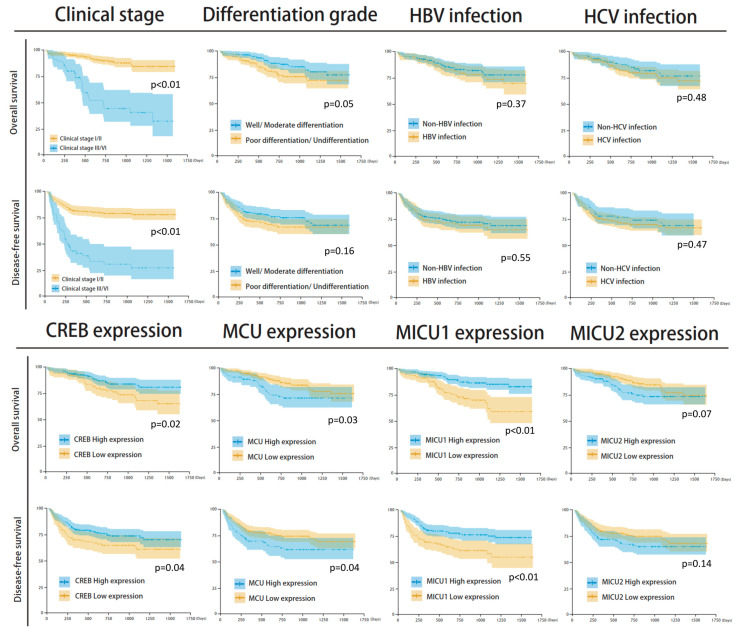
Kaplan–Meier analysis of clinicopathological parameters, including clinical stage, differential histologic grade, hepatitis B virus (HBV) infection, hepatitis C virus (HCV) infection, expression of CREB, MCU, MICU1, and MICU2 gene.

**Figure 5:**
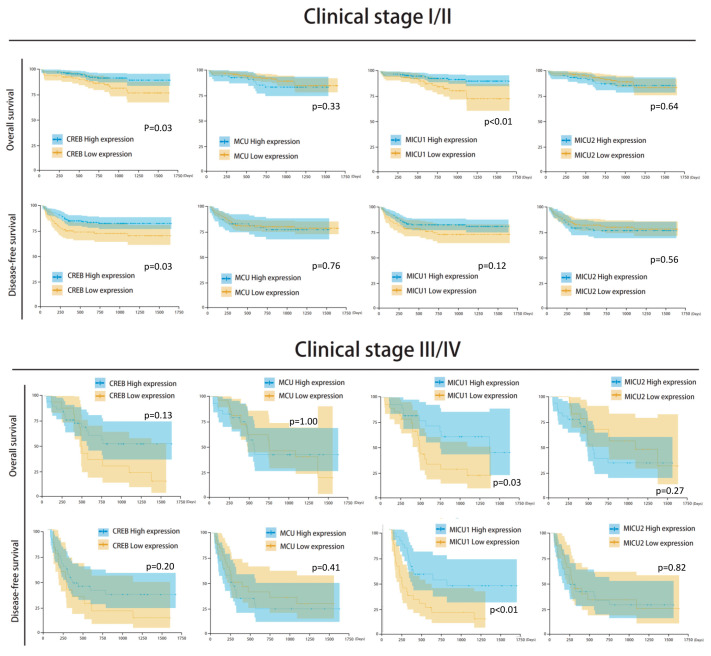
Kaplan–Meier analysis of the four mitochondria gene expression in clinical stage I/II and III/IV.

**Figure 6:**
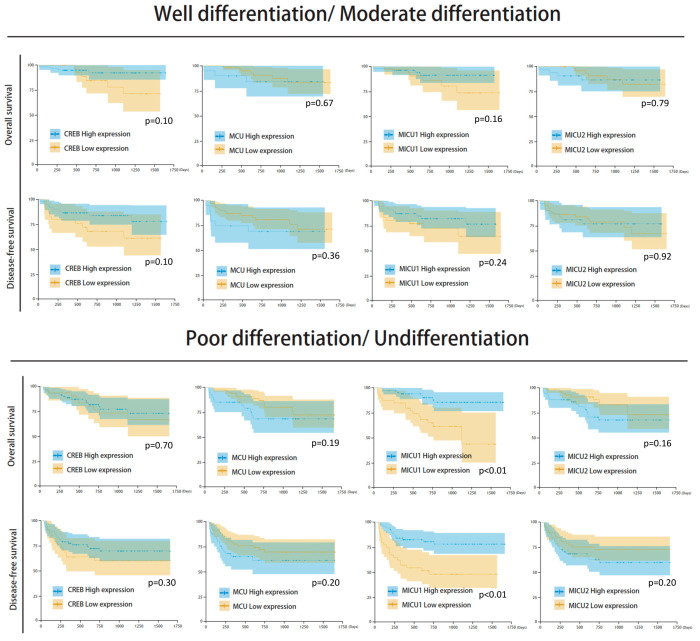
Kaplan–Meier analysis of the four mitochondria gene expression in well/moderate differentiation and poor differentiation/undifferentiation.

Among the clinicopathological parameters analyzed, differential histological grade and clinical stage were found to be significantly associated with the survival outcome. Differential histological grade analysis revealed that the median number of survival days in patients with well/moderate differentiation was 831 (survival rate = 84.9%) and with poor differentiation/undifferentiation was 732 (survival rate = 75.7%) (*p* = 0.05). Further, clinical stage analysis showed that the median number of survival days in stage I/II patients was 812 (survival rate = 89.2%), and in clinical stage III/IV patients it was 489 (survival rate = 50.0%) (*p* < 0.001) (Figure 4). Moreover, the expression of the four mitochondrial biomarkers was found to be significantly associated with survival outcome. The groups with low expression of *CREB* and *MICU1* and high expression of *MCU* and *MICU2* showed poor survival rate and poor prognosis in overall survival and disease-free survival analyses (Figure 5). The survival rate was higher in groups with high expression of *CREB* (86.3% vs. 74.2%, *p* = 0.027), low expression of *MCU* (84.9% vs. 75.7%, *p* = 0.033), high expression of *MICU1* (88.2% vs. 72.4%, *p* < 0.001), and low expression of *MICU2* (85.0% vs. 78.2%, *p* = 0.073). The Kaplan–Meier analysis revealed that groups with low expression of *CREB* and *MICU1*, and high expression of *MCU* and *MICU2* had fewer overall survival days (Figure 5). Similar results were observed in the Kaplan–Meier analysis of patients with high histological grade and clinical stage of HCC (Figure 5). In subgroup analyses, the four gene expressions impaired the clinical outcomes in differentiation type and clinical stage (Figure 6). The high expression of *MICU1* indicated a poor clinical outcome in the poor differentiation/undifferentiation group. Although there was no statistical significance in *MCU* and *MICU2*, a trend was observed in the poor differentiation/undifferentiation group. The *CREB* gene expression seemed more effective in the well/moderate differentiation group. These results suggest that the expression of mitochondria biomarkers is crucial for the survival outcomes in HCC patients.

**Figure 7:**
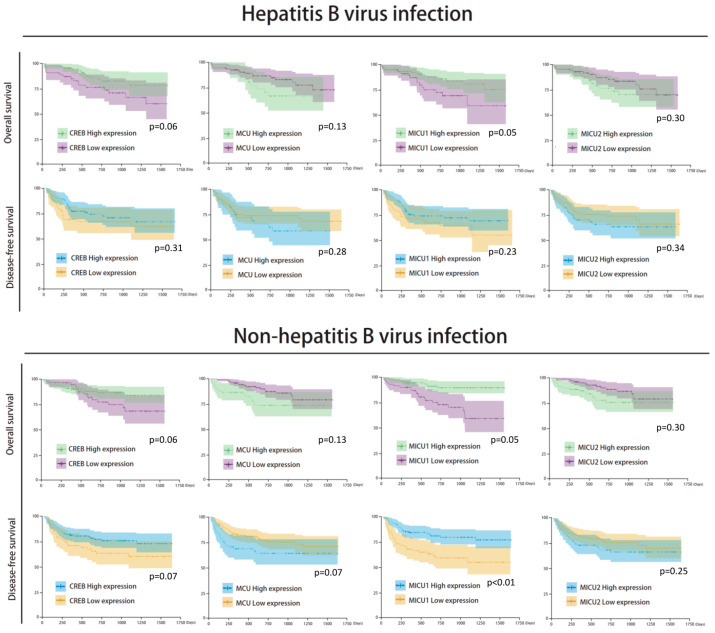
Kaplan–Meier analysis of four mitochondria gene expression in HBV/non-HBV infection subgroup analysis.

**Figure 8:**
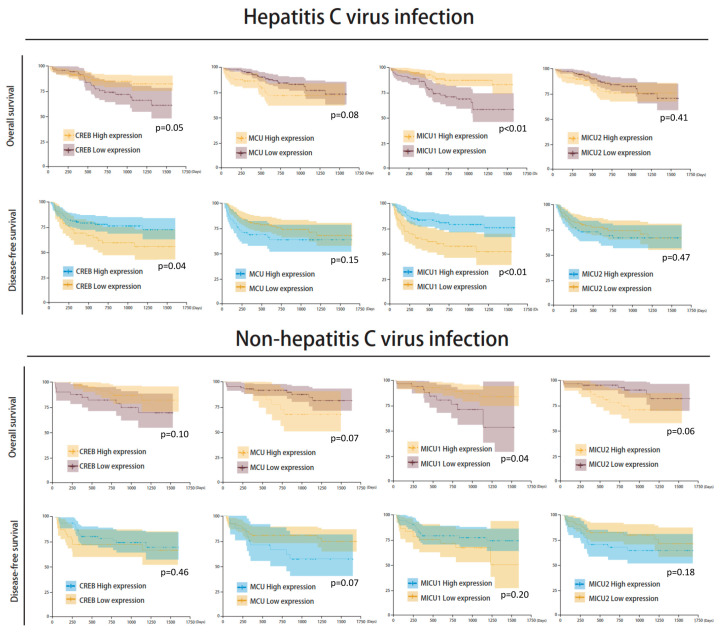
Kaplan–Meier analysis of four mitochondria gene expression in HCV/non-HCV infection subgroup analysis.

**Figure 9:**
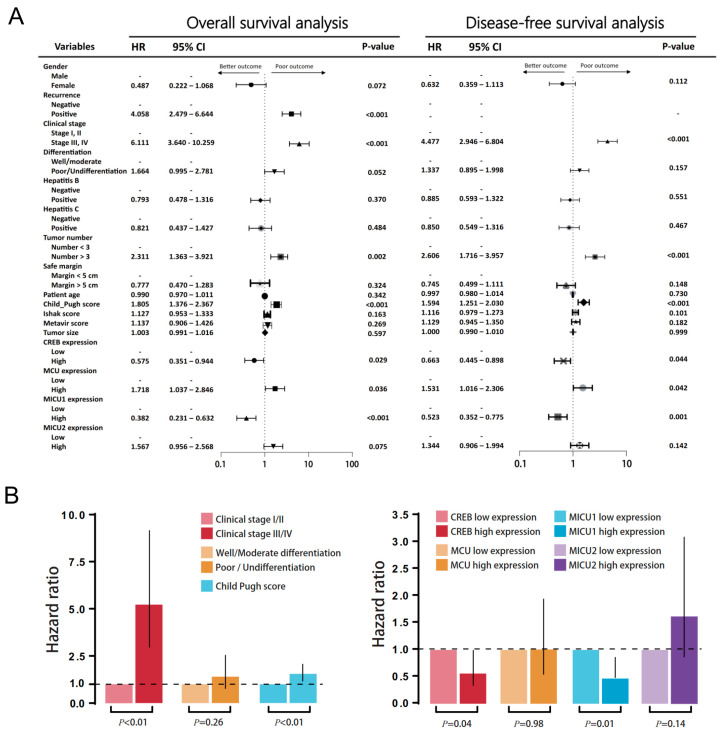
(**A**) Univariate analysis of influence of clinical characteristics in HCC patients. (**B**) Multivariate analysis of influence of clinical characteristics in HCC patients.

There was no significant correlation between mitochondrial biomarker expressions and HBV/HCV infection (Figure 7 and Figure 8). The survival analysis in both HBV and non-HBV infection groups showed the high expressions of *CREB* and *MICU1* were associated with better outcomes. In disease-free survival analysis, the mitochondria biomarker expression was insignificant, and there was no significant difference in the trend in the HBV infection group. In the non-HBV infection group, low expression of *MCU*, and high expression of *CREB* and *MICU1*, were associated with better disease-free survival (Figure 7). In the HCV infection group, the expression of *CREB* and *MICU1* was significantly associated with overall survival and disease-free survival outcome. In the non-HCV infection group, low expression of *MICU1* and high expression of MCU and MICU2 were associated with better overall survival (Figure 8). These results suggest that the expression of mitochondrial biomarkers play an important role in the overall survival and disease-free survival. In the overall survival outcome of Cox regression analysis, the recurrent status, high clinical stage, tumor number, Child-Pugh score, and four mitochondrial DNA gene expression had prognostic significance. A similar result was shown in the disease-free survival outcome (Figure 9A). In multivariate analysis, the clinical stage presented more prognostic significance than did the other parameters. On the other hand, the expression of *CREB* and *MICU1* had prognostic significance (Figure 9B).

## 4. Discussion

In this study, we used IHC to verify that the expression of mitochondrial proteins in liver cancer tissues is higher than that of the adjacent normal tissues, consistent with a previous report on HCC in the TCGA database. The role of the CREB protein as a promoter regulating the cancer cells has been previously confirmed in liver cancer cells (HepG2). It has been reported that the promoters of other members can be recognized by CREB, and once bound to the promoter, the tumor highly expresses MCU, MICU1, and MICU2. This study demonstrates that the mitochondrial genes CREB, MCU, MICU1, and MICU2 regulating mitochondrial Ca^2+^ uptake correlate with the survival rate of HCC patients. The poor prognosis of HCC is significantly associated with lower CREB/MICU1 and higher MCU/MICU2 in HCC tissues compared to normal tissues, providing a novel prognostic panel for clinical prediction. These findings suggest that modulating mitochondrial Ca^2+^ and targeting these mitochondrial genes may be potential novel therapeutic strategies for HCC.

The expression and functions of CREB have been found to be altered in various types of cancer, and such alterations affect the overall survival and response to therapy in tumor patients [19]. For example, CREB is overexpressed in hematopoietic tumors such as acute lymphoblastic leukemia (ALL), acute myeloid leukemia (AML), Hodgkin’s lymphoma, chronic lymphatic leukemia (CLL), as well as solid tumors such as melanoma, renal cell, ovarian, prostate, lung, gastric, esophageal, pancreatic, and breast carcinoma, brain tumors and HCC [20]. Yen et al. demonstrated that HCC patients with overexpressed CREB mRNAs show poor prognosis [21]. A CREB-associated pathway is involved in the pathogenesis and progression of HCC [22,23]. Thus, our data showing that overexpression of CREB may be involved in the poor prognosis of HCC conforms to the previous studies in the field. Our results were further confirmed by bioinformatic analyses showing that, in the process of tumor progression, the combination of MCU and MICU1/2 complexes promotes the expression of the three proteins. Interestingly, CREB can bind to the upstream promoter of MCU in HepG2 cells.

Several recent studies have contributed to our understanding of the molecular foundations of the MCU complex in the regulation of Ca^2+^ influx into mitochondria, as well as its implication in cancer progression [4]. The molecular mechanisms underlying the actions of the MCU complex involve the pore-forming molecule MCU and its regulatory subunits such as EMRE, MICU1, MICU2, and MCUb. Liu et al. demonstrated that the upregulation of MCU is associated with poor prognosis in colorectal cancer (CRC) patients, and MCU promotes mitochondrial Ca^2+^ uptake to enhance dephosphorylation of mitochondrial transcription factor A (TFAM), leading to mitochondrial biogenesis in CRC [24]. Furthermore, Ren et al. showed that HCC patients with high MCU or low MICU1 exhibit a poor survival rate, and MCU promotes metastasis by inducing ROS production [16]. In this study, we demonstrated that high MCU/MICU2 and low MICU1 are associated with poor prognosis, highlighting their significance in predicting clinical outcomes, further suggesting that the mitochondrial Ca^2+^ uptake machinery may be a potential therapeutic target for HCC. Nevertheless, a limitation of this study is the lack of validation experiments using gene manipulation that limits the elucidation of the relationship between this mitochondrial Ca^2+^ uptake machinery. Thus, further studies are required to decipher the biological role of mitochondrial Ca^2+^ uptake in the progression of HCC.

MCU is regulated at many levels, including the interaction of proteins and their regulatory components (MCUb, MICU, MCUR1, EMRE, and SLC25A23), transcriptional regulation of CREB and microRNA, as well as post-translational modification of oxidation and phosphorylation to Ca^2+^ and Mg^2+^ divalent cations [25]. MICU1 and MICU2 play a synergistic role in MCU regulation and together act as the gatekeepers of the channel in a complex manner [26]. Previous studies have suggested that under low cytoplasmic Ca^2+^ levels, MICU1 and MICU2 form a loose dimer, thereby inactivating MCU [27,28,29,30]. With the increase in the cytoplasmic and mitochondrial inter-membrane space of Ca^2+^ concentration, Ca^2+^ binds to MICU EF-hand and triggers a conformational rearrangement, thereby promoting a close interaction of MICU1/MICU2 and alleviating MCU inhibition. In addition, it does not affect the MICU2 levels, but it affects the mitochondrial Ca^2+^ uptake after the MICU1 gene is knocked down or overexpressed in liver cancer cells [31]. In addition, the elimination of MICU2 protein also inhibits the absorption rate of mitochondrial Ca^2+^ [32]. This may be due to the lack of MCU gatekeeping, which increases the resting Ca^2+^ level of the mitochondria.

Although the reasons why some cancer cells acquire diametrically opposed alterations in the mitochondrial Ca^2+^ dynamics are unknown, it is tempting to invoke the extraordinary metabolic and functional flexibility that generally accompanies malignant transformation as a main factor. Thus, while cancer cells that synthesize ATP by glycolysis may achieve increased resistance to cell death by MCU inhibition, malignant cells that primarily rely on mitochondrial respiration for ATP synthesis are expected to require a hyperactive MCU complex (upon MCU upregulation or MICU1 downregulation), calling for the establishment of alternative cytoprotective pathways.

Ca^2+^-dependent ROS generation also occurs when MICU1 is downregulated, reflecting the physiological role of MICU1 as an MCU inhibitor [33,34]. Accordingly, reduced MICU1 levels and high MCU:MICU1 ratios have been associated with poor disease outcomes in patients with hepatocellular carcinoma [16] and breast cancer [15], respectively. Notably, MCU, whose conductivity for Ca^2+^ is positively regulated by ROS-driven S-glutathionylation [35], also controls cell-cycle progression by generating spontaneous mitochondrial Ca^2+^ transients that coordinate mitotic entry supporting proliferation [36,37], thus suggesting yet another mechanism for ROS-driven alterations in mitochondrial Ca^2+^ fluxes to support tumor progression. The identification of the heritable mutations in the components of the MCU complex leading to disease underscores the importance of not only the MCU channel but also the diverse regulatory controls of MCU function.

## 5. Conclusions

This study demonstrates the prognostic significance of CREB, MCU, MICU1, and MICU2 in predicting the outcomes of HCC. Low levels of CREB/MICU1 and high MCU/MICU2 in HCC tissues are associated with poor prognosis, thus shedding light on novel potential strategies for the clinical management of HCC patients.

## Figures and Tables

**Table 1:** The relationship of clinicopathological characteristics with cyclic adenosine monophosphate response element-binding protein (CREB), mitochondrial calcium uniporter (MCU), mitochondrial calcium uptake (MICU)1 and MICU2 expression in hepatocellular carcinoma (HCC) patients.

		CREB Expression			MCU Expression			MICU1 Expression			MICU2 Expression		
Variables	Total	Low	High	*p*-Value	Low	High	*p*-Value	Low	High	*p*-Value	Low	High	*p*-Value
Case number, *n*	354	120	234		251	103		134	220		207	147	
Age	63.2 ± 11.5	63.8 ± 11.2	62.9 ± 11.6	0.50	64.5 ± 11.4	60.3 ± 11.2	0.02	63.5 ± 10.8	63.1 ± 11.9	0.77	64.5 ± 11.3	61.5 ± 11.5	0.02
Gender, *n*													
Male	285	99	186	0.57	202	83	1.00	110	175	0.58	167	118	1.00
Female	69	21	48		49	20		24	45		40	29	
Child-Pugh score	5.2 ± 0.7	5.3 ± 0.8	5.2 ± 0.7	0.36	5.2 ± 0.7	5.3 ± 0.9	0.07	5.4 ± 0.9	5.2 ± 0.6	<0.01	5.2 ± 0.6	5.3 ± 0.9	0.34
Ishak score	3.8 ± 1.6	3.6 ± 1.6	3.9 ± 1.6	0.20	3.8 ± 1.6	3.7 ± 1.7	0.59	3.6 ± 1.7	3.9 ± 1.6	0.05	3.6 ± 1.6	4.0 ± 1.6	0.02
Metavir score	2.7 ± 1.2	2.6 ± 1.2	2.8 ± 1.2	0.18	2.7 ± 1.2	2.7 ± 1.2	0.68	2.6 ± 1.2	2.8 ± 1.1	0.06	2.6 ± 1.2	2.9 ± 1.2	0.03
Hepatitis B, *n*	191	62	129	0.42	130	61	0.34	72	119	1.00	110	81	0.91
Hepatitis C, *n*	111	41	70	0.47	87	24	0.04	37	74	0.34	65	46	0.90
Survive, *n*	291	89	202	<0.01	213	78	0.05	97	194	<0.01	176	115	0.12
Recurrence, *n*	65	27	38	0.19	42	23	0.23	32	33	0.05	176	115	0.12
Survival days	796.8 ± 422.6	846.8 ± 447.7	771.2 ± 407.8	0.11	816.9 ± 420.5	747.8 ± 425.8	0.16	731.8 ± 395.6	836.4 ± 434.4	0.02	819.6 ± 408.1	764.7 ± 441.7	0.23
Clinical stage, *n*													
Stage I, II	298	103	195	0.65	224	74	<0.01	106	192	0.05	183	115	0.01
Stage III, IV	56	17	39		27	29		28	28		24	32	
Differentiation, *n*													
Well/Moderate	160	59	101	0.31	129	31	<0.01	62	98	0.83	107	53	<0.01
Poor/Undifferentiation	193	61	132		121	72		72	121		99	94	
Surgery, *n*													
Segmental resection													
One segment	93	25	68	0.10	72	21	0.11	30	63	0.21	55	38	0.90
Two segments	130	53	77	0.05	100	30	0.07	46	84	0.50	82	48	0.22
Three segments	56	19	37	1.00	38	18	0.63	21	35	1.00	32	24	0.88
Left lobectomy (LL)	9	2	7	0.72	4	5	0.13	1	8	0.16	3	6	0.17
Right lobectomy (RL)	20	4	16	0.23	14	6	1.00	8	12	0.82	12	8	1.00
Extended LL	28	11	17	0.54	14	14	0.02	18	10	<0.01	16	12	1.00
Extended RL	11	5	6	0.52	6	5	0.31	7	4	0.11	5	6	0.54
Others	7	1	6	0.43	3	4	0.20	3	4	1.00	2	5	0.13
Medicine, *n*													
Hepatitis	137	42	95	0.36	92	45	0.23	52	85	1.00	79	58	0.83

**Table 2:** Correlations among the total scores of CREB, MCU, MICU1, and MICU2 in HCC tissues.

Molecular Markers	*p*-Value			
	CREB	MCU	MICU1	MICU2
CREB	--	0.165 **	0.263 **	0.222 **
MCU	--	--	0.183 **	0.520 **
MICU1	--	--	--	0.386 **
MICU2	--	--	--	--

** A 2-sided *p* < 0.01 indicates statistical significance.
